# Total hip arthroplasty following failed fixation of proximal hip fractures

**DOI:** 10.4103/0019-5413.41851

**Published:** 2008

**Authors:** Shekhar Srivastav, Vivek Mittal, Shekhar Agarwal

**Affiliations:** Delhi Institute of Trauma and Orthopedics, Sant Parmanand Hospital, 18 Shamnath Marg, Delhi - 110 054, India

**Keywords:** Failed internal fixation, hip arthroplasty, hip fractures, THA

## Abstract

**Background:**

Most proximal femoral fractures are successfully treated with internal fixation but a failed surgery can be very distressing for the patient due to pain and disability. For the treating surgeon it can be a challenge to perform salvage operations. The purpose of this study was to evaluate the short-term functional outcome and complications of total hip arthroplasty (THA) following failed fixation of proximal hip fracture.

**Materials and Methods:**

In a retrospective study, 21 hips in 20 patients (13 females and seven males) with complications of operated hip fractures as indicated by either established nonunion or fracture collapse with hardware failure were analysed. Mean age of the patients was 62 years (range 38 years to 85 years). Nine patients were treated for femoral neck fracture, 10 for intertrochanteric (I/T) fracture and two for subtrochanteric (S/T) fracture of the hip. Uncemented THA was done in 11 cases, cemented THA in eight hip joints and hybrid THA in two patients.

**Results:**

The average duration of follow-up was four years (2-13 years). The mean duration of surgery was 125 min and blood loss was 1300 ml. There were three dislocations postoperatively. Two were managed conservatively and one was operated. There was one superficial infection and one deep infection. Only one patient required a walker while four required walking stick for ambulation. The mean Harris Hip score increased from 32 preoperatively to 79 postoperatively at one year interval.

**Conclusion:**

Total hip arthroplasty is an effective salvage procedure after failed osteosynthesis of hip fractures. Most patients have good pain relief and functional improvements inspite of technical difficulties and high complication rates than primary arthroplasty.

## INTRODUCTION

Due to increase in the aging population, the number of hip fractures in the elderly population is increasing. The management of these fractures ranges from conservative method to osteosynthesis and primary replacement arthroplasty. More and more of these fractures are treated surgically by osteosynthesis for better rehabilitation and early return to function. Various factors causing failure following osteosynthesis include osteoporosis, unstable fracture reduction or poor implant position.

Management of failed internal fixation of proximal hip fractures includes revision osteosynthesis or conversion total hip arthroplasty (THA). Total hip arthroplasty is generally accepted as the most successful salvage procedure for failure of these fixation devices.^1^ Conversion of failed hip surgeries to THA is indicated where the bone quality is poor, head is damaged due to previous internal fixation, poor bone stock, or limb shortening. Total hip arthroplasty in these patients may be difficult because of presence of previous implant, poor bone stock, scarred tissues and increased risk of infection. The purpose of this study is to evaluate the short term functional outcome, technical difficulties, complications associated with hip arthroplasty performed after failed fixation of proximal hip fractures.

## MATERIALS AND METHODS

Between 1994-2005, 21 hips [Table 1] in 20 patients (13 females and seven males) with a mean age of 62 years (range, 38 to 85 years) were treated at our institution with hip arthroplasty, after failed fixation of proximal hip fractures, as established by nonunion or implant failure. Records of the patients were retrieved from our computer database.

**Table 1:** Details of the patients of failed proximal hip fractures where conversion THA is performed.

Age/Sex	Diagnosis at injury	Date of injury	Primary operative procedure	Conversion THA type and date	Interval B/w injury and replacement	Total follow-up	Complication
54/F	# NOF (L)	Jan-91	Broken canulated hip screw	Charnley cemented THA Aseptic loosening, Revision uncemented THA with long stem May 2002	44 Mths	13 years	Revision THA
74/M	I/T # (R)	Nov-94	DHS, Double angle blade plate	Uncemented THA, 2000	5 yrs	7 years	Superficial Infection
45/F	# NOF(R)	Oct- 96	Canulated hip Screw	Cemented THA, Apr 97	6 Months	10 years	—
66/F	I/T #	April -97	Blade plate failed, DHS Fixation	Uncemented THA, May 05	8 years	2 years	—
84/F	S/T #	Nov-97	Blade plate	Cemented THA, Dec 05	8 years	2 years	—
62/F	# NOF	May-98	Canulated hip screw	Cemented THA, May 99	12 Months	8 years	—
68/F	# I/T(L)	May-00	DHS	Cemented THA Oct 2000	5 Months	7 years	—
38/M	# NOF(L)	Sep-00	DHS, Pauwel's osteotomy	Uncemented THA (June 01)	11 Months	6 years	Dislocation - Change of liner
68/F	# I/T(R)	Mar-01	DHS	Cemented THA June 02	15 Months	5 years	—
44/F	# NOF(R)	April-01	Canulated hip screw	Uncemented THA, May 05	4 years	2 years	—
80/F	S/T # (R)	Aug-01	DHS	Uncemented THA, Sep 03	25 Months	4 years	—
50/M	# NOF(R)	Feb-02	Canulated hip screw	Uncemented THA, Sep 02	7 Months	5 years	—
67/F	I/T # (R)	Mar- 02	DHS- Cut through	Uncemented THA, Dec 02	9 Months	5 years	Dislocation
53/F	# NOF(L)	April- 02	Canulated hip screw	Hybrid THA 03	16 Months	4 years	—
56/F	I/T # (L)	Jan-03	DHS, PFN	Uncemented THA, July 2004	18 Months	3 years	—
41/M	# NOF(L)	Jan-03	Pauwel's osteotomy + Double Barrel plate	Uncemented THA, Feb 2005	25 Months	2 years	Deep infection debridement, and antibiotics
58/M	# NOF(L)	March-03	CCS- Non union with broken screw	Uncemented THA, Jan 04	9 Months	4 years	—
85/M	# I/T(L)	May-03	DHS	Cemented THA, Oct 03	5 Months	4 years	—
64/M	I/T # (R)	March-05	PFN	Cemented THA, June 05	3 Months	2 years	Dislocation
75/F	I/T # (R)	May-05	DHS	Hybrid THA Dec 05	7 Months	2 years	—
71/F	I/T # (L)	June- 05	DHs	Cemented THA, Nov 05	5 Months	2 years	—

#NOF = Fracture neck of femur, I/T - Intertrochanteric, # - Fracture, DHS - Dynamic hip screw, S/T - Subtrochanteric, THA - Total hip arthroplasty, PFN - Proximal femoral nail, CCS - Canulated cancellous screw

Out of 21 hip fractures where conversion THA was done, 10 were intertrochanteric fractures, nine were fracture neck of femur and two were subtrochanteric fractures. In all cases primary reduction and fracture fixation was done within three weeks of sustaining the fracture. Four out of 21 cases had two surgeries before conversion THA was done.

In one hip, intertrochanteric fracture (case no. 2) was treated with dynamic hip screw (DHS) fixation which failed with loss of reduction. Osteotomy was done one year after the first surgery using double-angled barrel plate which too failed for which uncemented THA was done. The second case (case no. 15), a 56-year-old female with intertrochanteric left femur was treated with DHS fixation, which failed. Salvage operation with proximal femoral nail was done seven months after the first surgery. The fracture went into nonunion and conversion THA was done one year after the second surgery [Figure 2].

**Figure 1:**
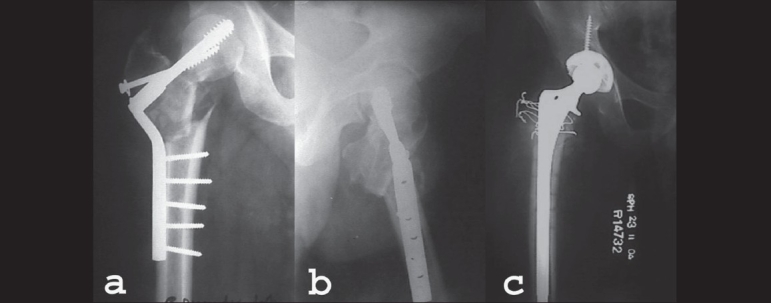
(a) AP radiographs of 38yrs old gentleman. Fracture neck of femur Rt.side treated with primary Powell's osteotomy and fixation with double angle barrel plate. (b) Lateral radiograph of same patient showing implants cutting out and fracture fragments in malaligned position. (c) AP radiograph showing uncemented total hip arthroplasty done 14 months after the primary fixation surgery

**Figure 2:**
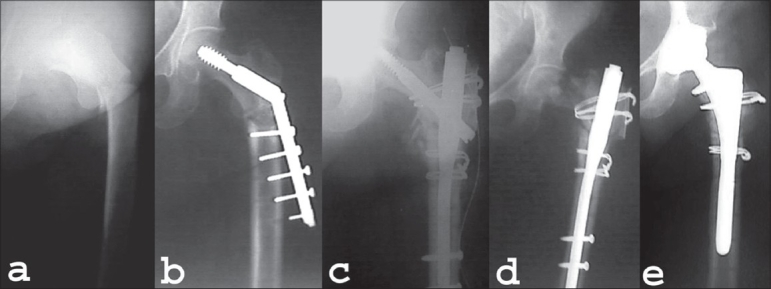
(a) AP radiograph of 58yrs old lady with subtrochanteric fracture of her left hip. (b) Fracture was fixed with DHS which pulled out in 4 months time. (c) DHS was removed and refixation was done with proximal femoral nail. (d) Leg screw of PFN cut through the head and was removed. Fracture went into non-union. (e) PFN removed and uncemented THA performed 20 months after fracture

In the third case (case no. 8), a 38-year-old male who suffered right femoral neck fracture and was treated initially with closed reduction and DHS fixation. On implant failure, valgus osteotomy and DHS fixation was done after four months. The fracture of neck of femur did not unite and hence conversion to uncemented THA was done six months after second surgery. In the fourth case (case no. 4), a 66-year-old female patient with intertrochanteric fracture of left femur was treated with blade plate fixation but fracture did not unite. Revision surgery was performed with DHS after four years and two months. The implant cut through the neck. Uncemented THA was done five years after the second surgery.

**Figure 3:**
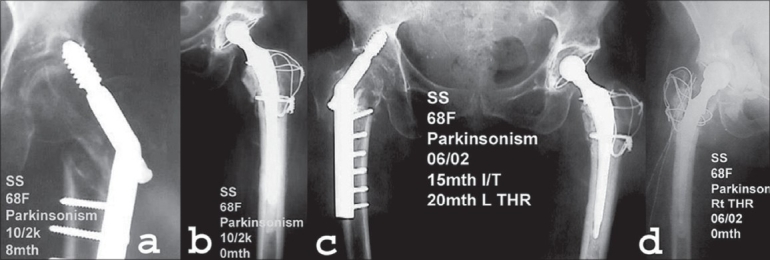
(a) AP radiographs of 68yr old lady with parkinsonism showing lag screw cut out of DHS done for intertrochanteric fracture of left hip done eight months back. (b) DHS was removed and cemented total hip arthroplasty with trochanteric wiring was performed. (c) AP radiograph of same patient showing failure of DHS fixation for intertrochanteric fracture of right hip fifteen months after the fracture. (d) DHS was removed and cemented total hip arthroplasty of right hip was performed

**Figure 4:**
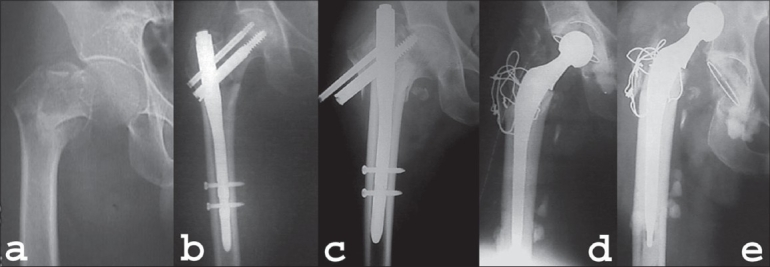
(a) AP radiograph of 70 year old patient showing intertrochanteric fracture of right hip. (b) Fracture was fixed with Proximal femoral nail. (c) Fracture collapse with screw back out three months after the surgery. (d) PFN was removed and cemented total hip arthoplasty was performed and trochanteric wiring was done. (e) Patient had dislocation of his hip two weeks after replacement surgery which was managed conservatively

Of the remaining seven intertrochanteric fractures, one patient (case no. 7) had fracture of both hips with a gap of one year [Figure 3]. She had Parkinson's disease. The fractures on both sides were fixed initially with DHS which cut through the head and conversion THA had to be performed. One patient with intertrochanteric fracture was fixed with proximal femoral nail [Figure 4]. The lag screws backed out within six weeks and fracture collapsed for which conversion THA was done. The remaining four patients with intertrochanteric fractures were treated with DHS where lag screws cut out occured through the femoral head and conversion THA was performed.

One fracture neck of femur was managed with primary Pauwell's osteotomy and fixation with double-angle barrel plate [Figure 1]. The fracture remained ununited at the end of one year. In other seven femoral neck fractures, fixation was done with cannulated hip screws and fracture went into nonunion. In two of these cases screws were broken.

One of the subtrochanteric fracture was treated with blade plate assembly where there was implant failure and plate lifted off the bone. Another subtrochanteric fracture was managed with DHS fixation where the lag screw backed out excessively with collapse and there was failure of fixation. In both the cases bones were very osteoporotic. Failure of primary fixation had also caused damage to the femoral head. Both the patients were more than 80 years of age. Considering their advanced age, it was felt that THA will give them a better chance of early ambulation.

All patients underwent preoperative detailed clinical examination and evaluated for medical co-morbidities. Patients' medical history, operative notes, discharge summaries and previous and fresh radiographs were retrospectively reviewed. Occult infection as a cause of failure is always considered and a complete preoperative blood count with differential determination of erythrocytes sedimentation rate and C-reactive protein was done.

### Surgical procedure

Total hip arthroplasty was performed by a team headed by the same surgeon. Cemented or uncemented THA was done as decided by the operating surgeon according to the age of the patient and according to condition of bones as seen in preoperative radiographs and also per-operatively. Cementless THA was the preferred choice. Physiological age and patients' level of activity were a major determinant. Younger and active patients were advised uncemented THA. In patients with defect in acetabulum, grafting and cemented THA was done. Uncemented THA was performed in 11 cases, cemented THA in eight cases and hybrid THA in two cases. An attempt was made to incorporate the previous scar in the incision but if not possible fresh incision was made. Transtrochanteric approach was used in those cases where trochanter was fractured or avulsed (n=11). Intraoperative specimen was sent for gram stain and AFB stain (in all cases) to rule out any infection. Implants were removed and bony defects in femur and acetabulum assessed. Autograft was used in five cases to fill up the bony defect in the acetabulum. Cemented or uncemented THA was performed depending on the bone quality, bone defects, patients' general condition and affordability. Replacement was done using standard technique and stability was checked before the closure of the wound. Failed subtrochanteric and intertrochanteric fractures were more challenging and difficult than femoral neck fracture. There was abnormal position of trochanter mass because of rotation defect and malunion of upper femur in frontal and sagittal plane. There was difficulty in intraoperative identification of limb length due to loss of usual landmarks such as lesser trochanter. Removal of fracture screws sometimes required use of a trephine and bridging the last screw hole with a longer stem. While implanting cemented stems screw holes were closed with either bone grafts or by assistant's finger. But there was some cement extrusion from the medial side. Attention was paid to maintain the integrity of abductor mechanism. Trochanter if detached was reattached with tension band wiring technique of Charnley (n=12).

Active assisted exercises were started during the first postoperative day and according to patient condition, ambulation started on second or third day. Patients were first ambulated with a walker, then with a stick and gradually progressed to ambulation without any support according to their recovery. Antibiotics were started on the day of surgery with first dose given preoperatively and continued till the third postoperative day. Dressing was changed on the third postoperative day and at the time of discharge on the fifth day. Stitches were removed on the 14^th^ day of the surgery.

Thereafter patient was reviewed at six weeks, three months, six months and at one year and yearly thereafter. Follow-up proforma was filled at every visit and clinical and radiological results were recorded at each visit.

The cement grade of the femoral stem was evaluated according to the criteria reported by Barrack, Mulroy, and Harris.^2^ Radiological loosening of the acetabular component was classified according to the criteria of DeLee and Charnley^3^ and those proposed by Hodgkinson *et al.*^4^ An acetabular component was considered to be loose if a continuous radiolucent line was evident in all three zones, or if the acetabular component migrated. Migration of an acetabular component was defined by a change in the opening angle of more than 8° or a difference in the component position of > 3 mm when comparable radiographs were compared. Fixation of the cementless femoral component was evaluated according to the criteria described by Engh *et al.*^5^,^6^ and was classified as “bone ingrowth”, “stable fibrous” or “unstable”. Loosening of the cemented femoral component was evaluated according to the criteria described by Harris *et al.*^7^,^8^ and was graded as “definite” “probable”, “possible” or none.

## RESULTS

The median duration between the primary surgery for fixation of fracture and THA was nine months (range 3 months – 8 years). The mean operating time for hip arthroplasty operation was 125 min (range 95 min to 210 min) which included the time to remove the retained hardware such as screws or barrel plates. The mean estimated blood loss was 1300 ml. In one patient difficulty was encountered in removing the screws from the plate and the screw heads had to be cut to remove the plate and extricate the screws. Three patients had intraoperative complications. In two patients the proximal femoral canal got fractured during reaming and was treated with circlage wires. One patient had fracture of the greater trochanter and wiring was done. Uncemented THA was performed in 11 cases, cemented THA in eight cases and hybrid THA was performed in two cases. Trohanteric wiring was done in 12 patients. All surgeries were performed in single stage.

Three patients had postoperative complications in the form of dislocation of the hip. All the dislocations were early. In one the dislocation was anterior, observed on the third postoperative day and was subsequently reduced under general anesthesia (GA) and maintained with careful and protected physiotherapy. There was no further dislocation afterwards. The second patient dislocated the head anteriorly on the first postoperative day and was reduced under GA but was unstable on the operation table. So surgery was done and the acetabular lip liner with 10 mm elevation was oriented anteriorly. No further dislocation occurred thereafter. In the third patient, the hip got dislocated during physiotherapy and was reduced under GA, but got redislocated again. It was managed conservatively with master hinge brace for six weeks after which there was no dislocation.

Infection was seen in two patients. The superficial infection in one case was treated with antiseptic dressings and antibiotics only. Second case developed deep infection eight months after THA. This was a case of fracture neck of femur where primary Pauwel's osteotomy with fixation with double-angle barrel plate was done. The patient was also a known diabetic. Wound was opened and debridement was done following which antibiotics were given, repeated dressings were done. Prosthesis was retained and infection was controlled in three months.

One patient (case no. 1) had aseptic loosening of Charnley cemented THA eight years after salvage arthroplasty and was revised to uncemented THA and it was stable at 63 months of follow-up. Four patients developed medical complications such as paralytic ileus (*n*=2), urinary tract infection (*n*=1), congestive heart failure (*n*=1). All recovered over a period of time. There was no intraoperative or postoperative mortality.

The average duration of follow-up was four years (range two years to 13 years). There was no mortality. The patients were followedup at the time of removal of skin stitches two weeks, six weeks, three months, six months, one year and then every year. There was dramatic pain relief in all the patients with four patients reporting moderate and three reporting mild pain. One patient was using a walker whereas four were using a walking stick for ambulation. The mean Harris Hip Score increased from 32 preoperatively to 79 postoperatively at one-year interval. We did not find any difference clinically between cemented and uncemented hips as regards to pain and function.

Radiographic follow-up of more than three years was done in 15 patients. In one patient there was aseptic symptomatic loosening of the cemented Charnley stem after eight years which was converted to uncemented stem. Stable non-progressive radioleucent lines were found around the cup in two cases and around the stem in one case. Clinically, the patients were asymptomatic in all these three cases.

## DISCUSSION

Although most fractures of the proximal femur are treated with a favorable outcome, a complication can result in ongoing hip pain and disability. The reported failure rate with internal fixation for intertrochanteric fracture is in the range of 3-12% with device penetration (2-12%), nonunion (2-5%) and malunuion causing varus deformity (5-11%).^9^ In displaced intracapsular hip fractures 20-36% of patients initially treated with reduction and internal fixation required revision within two years usually because of nonunion or avasular necrosis.^10^ Parker *et al.*, also showed a reoperation rate of 40% for displaced femoral neck fracture treated with internal fixation.^11^

Total hip arthroplasty is generally accepted as the most successful salvage procedure for failure of these fixation devices.^1^ Hip arthroplasty dramatically alleviated pain and improved function in the majority of these patients, for whom other salvage techniques would have been difficult or had been tried and had failed. The operation allowed most patients to regain function that otherwise had been lost, which is the hallmark of an effective salvage procedure.

The surgeon who is faced with failed internal fixation of a proximal hip fracture should always consider occult infection as a potential cause of the failure. Our current protocol involves a complete preoperative blood count with differential determination of the erythrocyte sedimentation rate and C-reactive protein level. If there is evidence of infection, all hardwares are removed, irrigation and debridement is performed and the arthroplasty is performed in a staged fashion after the intravenous administration of organism-specific antibiotics. In our series there was no evidence of infection as a cause of failure of internal fixation in any of the cases and all surgeries were performed in single stage.

Failed internal fixation devices, frequently with broken screws, must be removed from the femur. Special instruments for the removal of broken screws can simplify this process. The surgery takes a longer time because the internal fixation device must first be removed. The surgeon must dissect through the old scars to expose the internal fixation device. This also causes increased blood loss. The ununited head and neck fragment or fragments usually are in a deformed position and must be mobilized before being excised.

Many specific problems may occur during conversion of failed internal fixation of intertrochanteric fractures to hip arthroplasty. The anatomy of the proximal femur usually is distorted, especially if the reduction of the hip fracture is imperfect, or if there is communition of the medial bony buttress. The bone quality usually is poor as a result of preexisting osteoporosis, which further decreases as a result of disuse after the failure of internal fixation. The greater trochanter either is not solidly healed or can be fragmented again during hip arthroplasty, thus affecting the abduction function, which leads to an increased dislocation rate and can adversely affect the ambulatory function. In our series there were three dislocations, out of which one was managed surgically and the other two were managed conservatively. Mabry *et al.*, showed a dislocation rate of 9% for secondary total hip arthroplasty.^12^ A high dislocation rate (6% for total hip replacement and 12% for hemiarthroplasty) has been demonstrated in other series in which THA was performed for the treatment of nonunion at the site of a femoral neck fractures.^13^ Proper reattachment of trochanter with either tension band wiring or trochanteric plate is necessary for the stability of the hip and proper functioning of the abductor mechanism.

One difficulty encountered in intertrochanteric fractures is containment of cement when it was being pressurized into the femoral medullary canal. The lag screw hole can be closed by the assistant's thumb, by firmly packed gauze, by a surgical glove inflated with saline or by fashioning a bone plug from the femoral head.^14^ For the screw holes, one could apply direct finger pressure, use gauze or, screws that were cut short to close the holes over the lateral cortex when cement is injected.^15^

Complication rates in conversion THAs are more than that seen in primary THAs. Infection rates generally increase in already operated areas and with additional hardware.^16^ The combined reported complications from published series are deep infection rate of 3.8%, periprosthetic fracture rate of 6.2%, dislocation rate of 11.4%, early implant failure rate of 1.5% and a reopertaion rate of 10.9%.^9^,^17^–^22^ These complication rates are higher than we would normally see in an osteoarthritic population undergoing primary THA. In our series also there was a deep infection rate of 4.76%, reoperation rate of 4.76% and dislocation rate of 14.28% which is comparable to other studies of a similar nature.

Several authors have found salvage THA for failed intertrochanteric fractures to be more difficult with a higher potential for complications than salvage THA for failed femoral neck fracture. Despite this finding, Haidukewych and Berry^17^ reported relatively few complications and good pain relief and function in their large series of salvage THA after failed IT fractures. By contrast, McKinley and Robinson^18^ reported poor outcomes in their series of salvage THA for failed subcapital fractures. Our subanalysis of salvage THA for failed internal fixation for intertrochanteric fractures and intracapsular neck fracture did not demonstrate any difference in complication rate or clinical outcome. The majority of our patients had good pain relief and marked functional improvement. In a few patients with residual hip pain, the most common apparent cause was trochanteric nonunion or trochanteric bursitis. Hip arthroplasty performed after failed internal fixation of proximal hip fractures is technically more difficult than routine primary THA. But despite the technical challenges hip arthroplasty is an effective salvage procedure after failed fixation of proximal hip fractures.
